# Supplementary material for: Adhesion of Bis-Salphen-Based Coordination Polymers to Graphene: Insights from Free Energy Perturbation Study
Source: Polymers (Basel). 2022 Oct 26;14(21):4525. doi: 10.3390/polym14214525 (PMC9657960; doi:10.3390/polym14214525)
Supplement: Supplementary file 1 [file polymers-14-04525-s001.zip › polymers-1858655-supplementary.pdf]

polymers

MDPI

Supporting Information

Adhesion of bis-salphen based coordination polymers to graphene: insights from free energy perturbation study

Sergey Pyrlin [1,*], Veniero Lenzi [1], Alexandre Silva[1], Marta Ramos [1] and Luís Marques [1]

[1] Physics Center of Minho and Porto Universities (CF-UM-UP), University of Minho, Campus de Gualtar, 4710-057 Braga, Portugal
* Correspondence: pyrlinsv@fisica.uminho.pt

S1. Impact of λ-schedule on FEP results for immersion of hydroxyl and carboxyl functional groups.

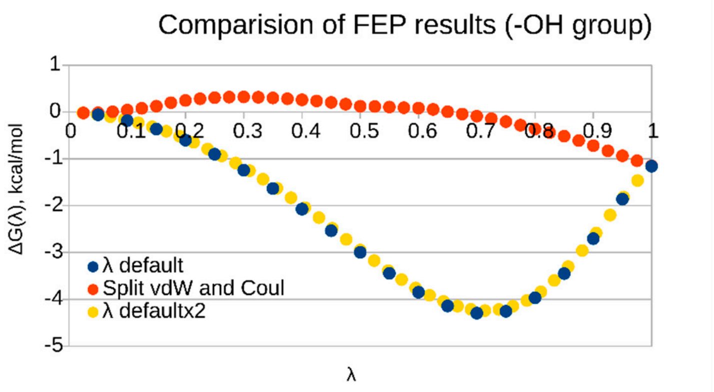

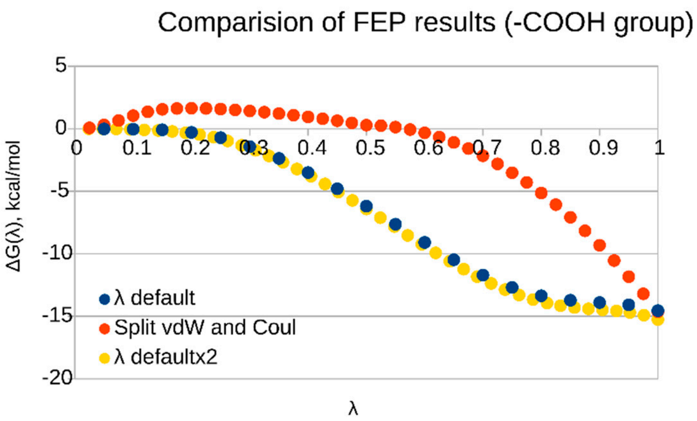

Figure S1: Accumulated change of Gibbs free energy for immersion of hydroxyl (top) and carboxyl (bottom) functional groups for 3 variants of λ-schedules: blue - default, used in FEP simulations with functional groups; yellow same as default, but with twice as much λ-points each simulated for twice as long time; red – simulation with van der Waals (λ<0.5) and Coulomb (λ≥0.5) interactions scaled consequently

S2. Radial pair distribution functions for compounds interaction with DCM solvent without and with graphene.

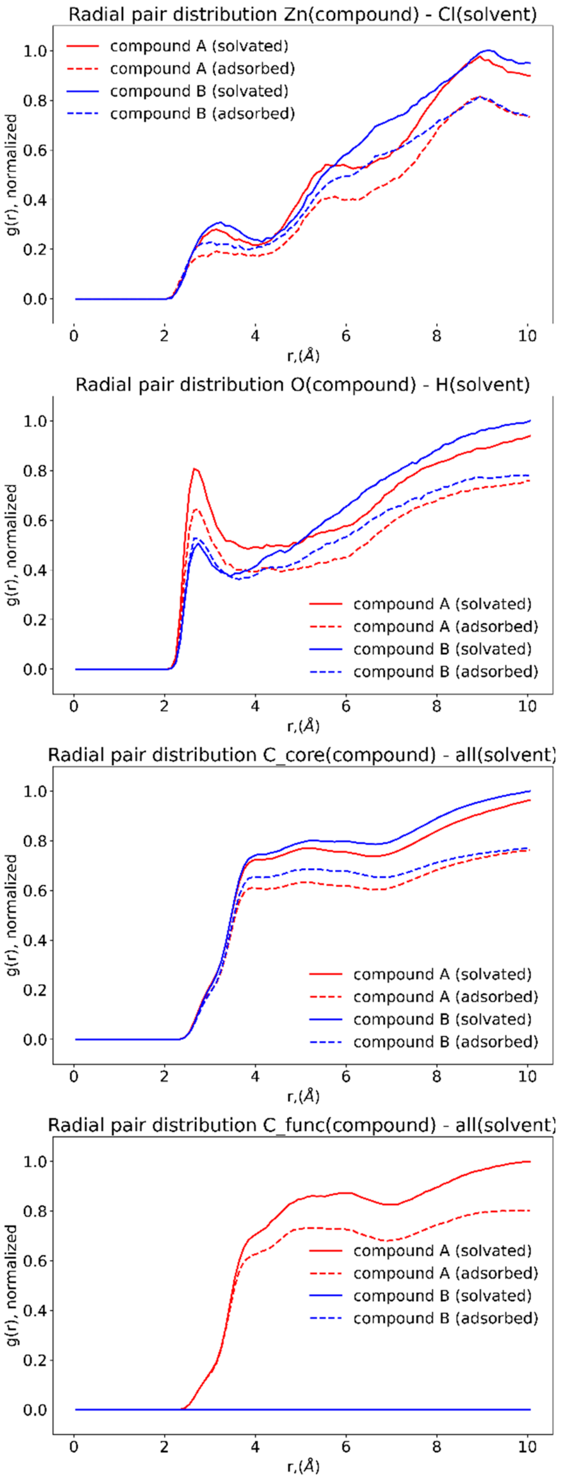

Figure S2: Radial pair distribution for cases (top to bottom): zinc cation - DCM chlorine, salphen oxygen - DCM hydrogen, carbon atoms of the bis-salphen ligand - any atom of solvent, carbon atoms of the functional phenyl rings - any solvent atom.

S3. Atomic partial charges used in MD simulations.

S3.1 Compound A: bis-(Zn)salphen with phenyl functional groups

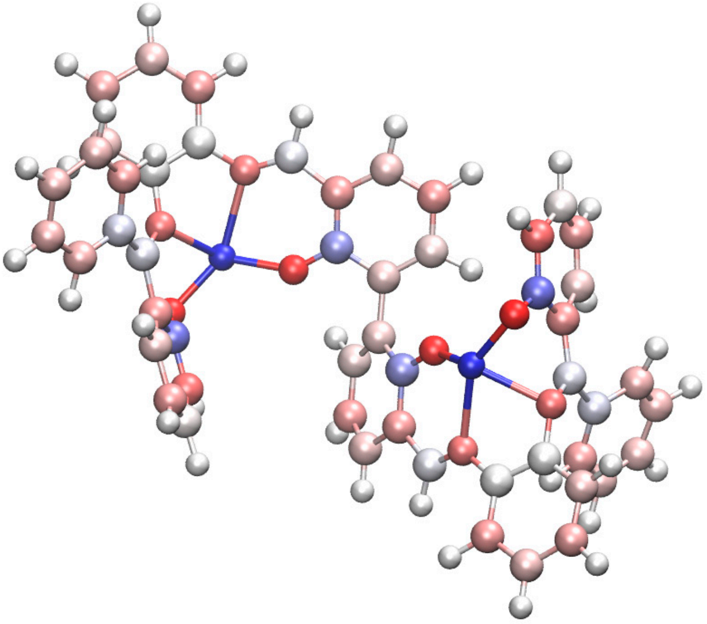

Figure S3: *Partial charge distribution for compound A. Blue - positive, red - negative.*

96

#	element	X	X	Z	q
	C	-0.099577	0.27387	-3.416637	-0.281215
	C	0.387856	-0.468019	-2.358131	0.563663
	C	1.180567	0.167322	-1.36854	-0.097838
	C	1.551636	1.512493	-1.517757	-0.065786
	C	1.10301	2.277054	-2.576649	-0.294408
	C	0.264315	1.650316	-3.472876	-0.123668
	O	0.057476	-1.685909	-2.249708	-0.693817
	C	-1.029554	-0.205873	-4.452612	0.254604
	N	-1.449112	-1.387138	-4.570665	-0.407049
	C	-2.63843	-1.73971	-5.217073	0.18542
	C	-3.008829	-1.111256	-6.401993	-0.270405
	C	-4.170895	-1.490765	-7.08174	-0.124425
	C	-4.847921	-2.682581	-6.675043	-0.225388
	C	-4.465149	-3.28878	-5.43717	-0.119346
	C	-3.432747	-2.745604	-4.680832	0.125776
	N	-3.079512	-3.168039	-3.368734	-0.432818
	C	-3.928865	-3.310629	-2.344273	0.272927
	C	-3.216902	-3.833076	-1.097244	-0.299583
	C	-3.848772	-3.629423	0.163908	-0.102201
	C	-3.262117	-3.981451	1.410761	-0.272004
	C	-2.027294	-4.547283	1.341434	0.003776
	C	-1.383404	-4.709462	0.107342	-0.462105
	C	-1.921577	-4.379278	-1.09753	0.700876
	O	-1.361959	-4.663412	-2.159613	-0.737265

C	-5.364025	-2.85409	-2.361969	0.301276
C	-6.307867	-3.55707	-1.632547	-0.184438
C	-7.698957	-3.398464	-1.739889	-0.168065
C	-8.232309	-2.478861	-2.613665	-0.143869
C	-7.333488	-1.635859	-3.376648	-0.140552
C	-5.9667	-1.920843	-3.247997	-0.253105
Zn	-0.879873	-3.068308	-3.093081	0.981684
H	2.276527	2.058229	-0.888931	0.133756
H	1.448195	3.318819	-2.801931	0.154764
H	-0.063206	2.439039	-4.205162	0.144291
H	-1.327732	0.587961	-5.127132	0.095978
H	-2.425848	-0.255806	-6.783781	0.167365
H	-4.424536	-1.087587	-8.090818	0.14949
H	-5.655897	-3.049084	-7.302869	0.1617
H	-5.117781	-4.084789	-4.978031	0.1297
H	-4.83103	-3.167738	0.132717	0.137485
H	-3.792049	-3.838228	2.323992	0.147492
H	-1.588043	-4.780808	2.35741	0.1362
H	-0.448336	-5.295518	0.024633	0.196153
H	-6.004225	-4.359316	-0.913527	0.134804
H	-8.277036	-4.154509	-1.257208	0.152029
H	-9.307966	-2.445171	-2.731827	0.147261
H	-7.647745	-0.878256	-4.084942	0.153005
H	-5.299925	-1.309601	-3.761814	0.167875
C	1.862679	-0.903362	2.285547	-0.281215
C	1.41312	-0.10154	1.176468	0.563663
C	1.531342	-0.642241	-0.139351	-0.097838
C	2.118567	-1.93631	-0.329088	-0.065786
C	2.516054	-2.729677	0.756395	-0.294408
C	2.189148	-2.283627	2.067072	-0.123668
O	1.212511	1.116367	1.333746	-0.693817
C	2.031069	-0.384428	3.655748	0.254604
N	1.813587	0.846025	3.965761	-0.407049
C	2.048449	1.489185	5.145649	0.18542
C	2.072342	0.797701	6.41272	-0.270405
C	2.386351	1.536331	7.591322	-0.124425
C	2.605791	2.893997	7.50597	-0.225388
C	2.613026	3.568241	6.293032	-0.119346
C	2.356066	2.836481	5.133732	0.125776
N	2.284914	3.477179	3.888931	-0.432818
C	3.170418	3.602283	2.901665	0.272927
C	2.709457	4.139008	1.579472	-0.299583
C	3.52454	4.250744	0.397881	-0.102201
C	3.073879	4.893984	-0.698967	-0.272004
C	1.844999	5.458954	-0.780352	0.003776
C	0.974148	5.157059	0.2255	-0.462105
C	1.393517	4.488174	1.410173	0.700876
O	0.48864	4.251539	2.213289	-0.737265
C	4.625343	3.122211	3.067068	0.301276
C	5.667439	3.851444	2.394403	-0.184438
C	6.988775	3.350903	2.363853	-0.168065
C	7.327442	2.230097	3.089113	-0.143869
C	6.319448	1.620399	3.866552	-0.140552
C	5.027151	2.071651	3.860125	-0.253105

Zn	0.815372	2.393591	2.605585	0.981684
H	2.257433	-2.421999	-1.317354	0.133756
H	3.059736	-3.601864	0.589949	0.154764
H	2.477259	-2.871357	2.918478	0.144291
H	2.382923	-1.162611	4.304386	0.095978
H	1.823652	-0.295658	6.51405	0.167365
H	2.41745	1.045448	8.553493	0.14949
H	2.749146	3.382418	8.448692	0.1617
H	2.519917	4.644152	6.279196	0.1297
H	4.435654	3.750889	0.285088	0.137485
H	3.793169	4.961004	-1.576305	0.147492
H	1.556108	5.958191	-1.714106	0.1362
H	-0.07762	5.45215	0.189791	0.196153
H	5.458235	4.813173	1.956343	0.134804
H	7.798876	3.938227	1.886402	0.152029
H	8.364893	1.950081	3.088938	0.147261
H	6.530464	0.852901	4.614172	0.153005
H	4.344666	1.505232	4.415764	0.167875

S3.2 Compound B: bis-(Zn)salphen without phenyl functional groups.

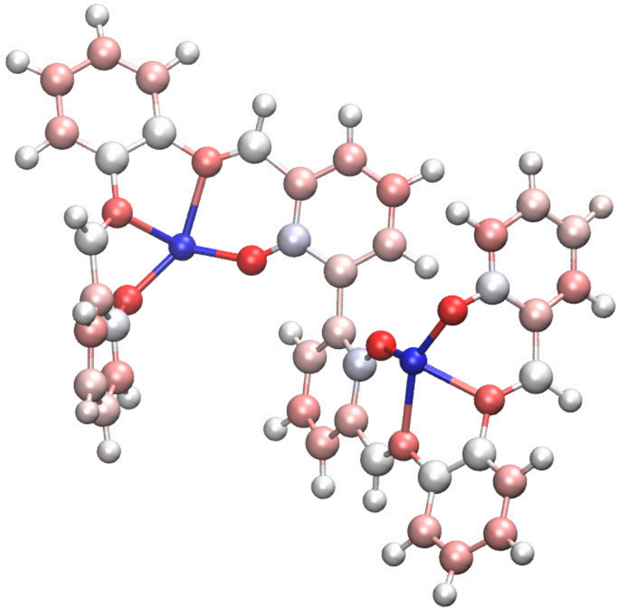

Figure S4: *Partial charge distribution for compound B. Blue - positive, red - negative.*

76

#	element	X	X	Z	q
	C	-0.251642	2.7757	-2.127558	-0.130227
	C	0.0224	1.393145	-1.966158	0.438758
	C	0.995119	1.049744	-0.947142	-0.025703
	C	1.651712	2.079588	-0.233545	-0.084391
	C	1.331839	3.389297	-0.421278	-0.27052
	C	0.417841	3.759203	-1.384184	-0.183891
	O	-0.493748	0.568836	-2.702845	-0.716034

C	-1.370136	3.26672	-2.974651	0.144319
N	-2.055079	2.509809	-3.748301	-0.380768
C	-3.21302	2.805309	-4.479568	0.152622
C	-3.532156	4.044949	-5.139307	-0.15389
C	-4.664602	4.2132	-5.878124	-0.182639
C	-5.583042	3.192179	-6.061667	-0.195321
C	-5.279707	1.985359	-5.491783	-0.155355
C	-4.148211	1.799562	-4.672395	0.124058
N	-3.837364	0.554653	-4.071001	-0.47105
C	-4.656815	-0.285866	-3.479284	0.232215
C	-4.098682	-1.686131	-3.159515	-0.102641
C	-4.578453	-2.479048	-2.143837	-0.237615
C	-4.115711	-3.764191	-2.033472	-0.045198
C	-3.074072	-4.215153	-2.769758	-0.037876
C	-2.471767	-3.319197	-3.664738	-0.241693
C	-2.975098	-2.115852	-3.857937	0.349283
O	-2.346123	-1.394531	-4.646661	-0.641663
H	-5.581526	0.073641	-3.004244	0.113366
Zn	-1.688997	0.313326	-4.100669	1.146396
H	2.453766	1.854748	0.484896	0.129015
H	1.95606	4.183615	0.013929	0.152182
H	0.035263	4.78857	-1.447693	0.149243
H	-1.601318	4.317905	-2.810514	0.128597
H	-2.932636	4.929419	-4.949559	0.133025
H	-4.820095	5.050201	-6.536728	0.160509
H	-6.417339	3.294199	-6.771271	0.160727
H	-5.897058	1.09791	-5.66504	0.142902
H	-5.318586	-2.137787	-1.507332	0.164645
H	-4.41964	-4.439873	-1.260752	0.021541
H	-2.638483	-5.14872	-2.570751	0.091587
H	-1.604822	-3.676775	-4.233811	0.121485
C	1.854956	-2.394238	0.526144	-0.130227
C	1.559443	-1.007019	0.49625	0.438758
C	1.469185	-0.401613	-0.818224	-0.025703
C	1.608673	-1.210379	-1.970335	-0.084391
C	1.909853	-2.534533	-1.876476	-0.27052
C	1.997952	-3.150509	-0.646761	-0.183891
O	1.331289	-0.39949	1.529428	-0.716034
C	2.18766	-3.105036	1.788597	0.144319
N	2.068924	-2.576271	2.949154	-0.380768
C	2.478918	-3.070431	4.194395	0.152622
C	2.456833	-4.440876	4.636181	-0.15389
C	2.824587	-4.812778	5.894375	-0.182639
C	3.229856	-3.891132	6.846222	-0.195321
C	3.20883	-2.57468	6.472452	-0.155355
C	2.892569	-2.166283	5.161192	0.124058
N	2.881344	-0.805063	4.767426	-0.47105
C	3.753414	0.131821	5.067154	0.232215
C	3.351339	1.582054	4.735966	-0.102641
C	4.261341	2.584364	4.495628	-0.237615
C	3.80747	3.869724	4.355045	-0.045198
C	2.488644	4.156279	4.259731	-0.037876
C	1.583462	3.086477	4.311337	-0.241693
C	2.000433	1.859327	4.554134	0.349283

O	1.120625	0.985327	4.546446	-0.641663
H	4.800001	-0.126204	5.286404	0.113366
Zn	1.247276	-0.521599	3.380327	1.146396
H	1.462118	-0.797222	-2.979206	0.129015
H	1.854551	-3.183113	-2.763203	0.152182
H	2.386742	-4.174337	-0.543736	0.149243
H	2.619047	-4.09095	1.623344	0.128597
H	2.270288	-5.236973	3.922646	0.133025
H	2.624434	-5.788126	6.303194	0.160509
H	3.388147	-4.183859	7.894678	0.160727
H	3.418985	-1.77216	7.186953	0.142902
H	5.276506	2.387989	4.478889	0.164645
H	4.444345	4.704151	4.144987	0.021541
H	2.16453	5.121393	4.005964	0.091587
H	0.517349	3.318348	4.197535	0.121485

S3.2 Hydroxyl functional group attached to grapehene.

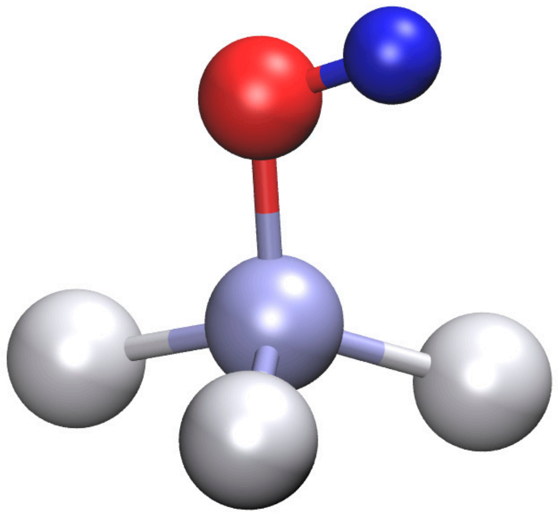

Figure S5: *Partial charge distribution for hydroxyl functional group. Blue - positive, red - negative.*

6

#	element	X	X	Z	q
	C	-0.091667	-0.045002	-0.313333	0.15
	O	-0.141668	-0.164999	1.096667	-0.585
	H	0.528332	0.395 1.466667	0.435	
	C	-1.331669	-0.775002	-0.743333	0
	C	-0.091667	1.385 -0.773333	0	
	C	1.128332	-0.794998	-0.733333	0

S3.2 Carboxyl functional group attached to grapehene.

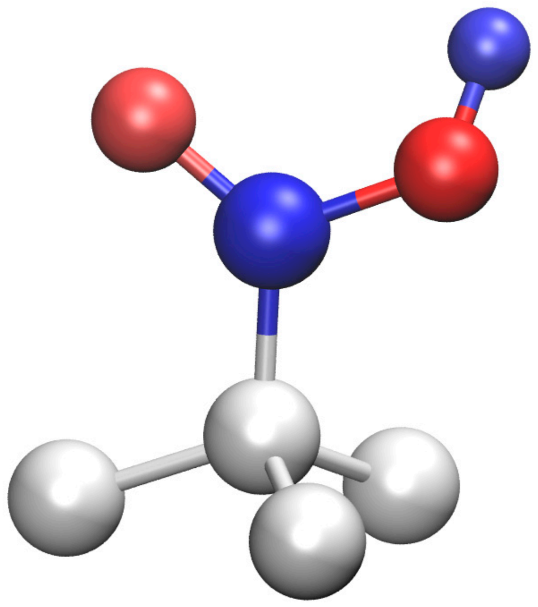

Figure S6: *Partial charge distribution for carboxyl functional group. Blue - positive, red - negative.*

8

#	element	X	X	Z	q
	C	-0.1525	-0.150003	-0.89125	0
	C	-0.1325	-0.190002	0.61875	0.52
	O	0.8675	0.559998	1.16875	-0.53
	O	-0.8925	-0.810003	1.35875	-0.44
	H	0.797501	0.989998	2.01875	0.45
	C	-1.3825	-0.890003	-1.48125	0
	C	-0.182501	1.319998	-1.35125	0
	C	1.077499	-0.830002	-1.44125	0
